# Current knowledge of morbidities and direct costs related to diabetic foot disorders: a literature review

**DOI:** 10.3389/fendo.2023.1323315

**Published:** 2024-01-17

**Authors:** Felix W. A. Waibel, Ilker Uçkay, Laura Soldevila-Boixader, Christina Sydler, Karim Gariani

**Affiliations:** ^1^ Orthopaedic Surgery, Balgrist University Hospital, University of Zurich, Zurich, Switzerland; ^2^ Infectiology, Balgrist University Hospital, University of Zurich, Zurich, Switzerland; ^3^ Infectious Diseases, Internal Medicine Department, Consorci Sanitari Integral-CSI, Sant Joan Despí Hospital, Barcelona, Spain; ^4^ Service of Endocrinology, Diabetes, Nutrition, and Therapeutic Education, Faculty of Medicine, Geneva University Hospitals, Geneva, Switzerland; ^5^ Diabetes Center of the Faculty of Medicine, University of Geneva Medical School, Geneva, Switzerland

**Keywords:** diabetic foot syndrome, infection, morbidity, cost, review

## Abstract

Diabetes is a chronic disease associated with numerous complications including diabetic foot disorders, which are associated with significant morbidity and mortality as well as high costs. The costs associated with diabetic foot disorders comprise those linked to care (direct) and loss of productivity and poor quality of life (indirect). Due to the constant increase in diabetes prevalence, it is expected that diabetic foot disorder will require more resources, both in terms of caregivers and economically. We reviewed findings on management, morbidity, mortality, and costs related to diabetic foot disorder.

## 1 Introduction

Diabetes mellitus is a major public health concern with a rapidly increasing prevalence over the past several decades. Its worldwide prevalence is estimated to be approximately 10.5%, representing 536.6 million people, with a projected increase in 2045 to 12.2%, representing 783.2 million individuals. The greatest increase in prevalence is expected in areas currently undergoing an economic transition from low to middle-income levels (1). Several factors contribute to the current increase in the prevalence of type 2 diabetes, including a sedentary lifestyle, unhealthy diet, population aging, urban expansion, and economic growth (1). Diabetes mellitus is a leading cause of mortality, decreased life expectancy, and reduced quality of life worldwide. In the presence of diabetes, all-cause mortality is estimated to increase by two to three times (2, 3). Among the different anatomical complications of diabetes and associated metabolic syndromes, diabetic foot disorders are the most recurrent, and they represent an ever-increasing health care problem.

In this narrative review, we discuss the current knowledge on the impact of diabetes-related (foot) complications, including diabetic foot disorders, in terms of quality of life and direct costs related to its prevention and therapies. However, in this review targeting healthcare workers in resource-rich countries, we deliberately did not address the associated costs and burden in terms of economical, psychological, epidemiological, societal, lifestyle, or political aspects, for which specific literature is available (4, 5).

### 1.1 Diabetic foot management

A diabetic foot ulceration (DFU) multidisciplinary team approach is highly recommended and has been shown to be the most effective strategy for reducing the rates of amputation and mortality in diabetic foot disorders (6, 7). Ideally, this team should include a diabetologist, podiatrist, infectious disease specialist, orthopedic surgeon, vascular surgeon, physiotherapist orthotist, diabetes nurse, and an interventional radiologist (8, 9). The team’s skills include wound care such as surgical and non-surgical wound debridement, adapted exudate control, vascular assessment, off-loading treatment, glycemia, lipid profile and blood pressure control, vascular and infection assessment, and, if required, revascularization procedures, antibiotic administration, and eventually amputation. The most important aspect of the DFU multidisciplinary team is that it comprises individuals with medical and surgical disciplines. Moreover, larger teams benefit from having a team leader and a team member structure, and clear referral pathways and care algorithms are important (10).

Wound debridement is performed to remove nonviable tissues that can interfere with wound healing by facilitating bacterial colonization and infection. Off-loading is a cornerstone of DFU management, as it allows redistribution of plantar pressure, promoting the healing process of DFU (11). The weight is then displaced to nearby areas that are not injured, thereby facilitating the healing process. Although offloading is fundamental, it can cause significant restrictions in daily life, mainly because of the resulting reduced physical movement.

In the presence of arterial insufficiency with hemodynamic impairment, a revascularization procedure that can be performed using an endovascular approach with angioplasty and stenting or surgical bypass grafting, or combination of these two if necessary, should be considered. Controlling plasma glucose levels can be beneficial, as hyperglycemia has been shown to be associated with delayed wound healing (12). The choice of anti-diabetic treatment must be individualized according to several parameters such as glycemic and weight goals, cardio-renal protection, side effects associated with treatments, mode of administration or even cost or accessibility (13). Stopping smoking is also beneficial for wound healing of DFU and must therefore always be considered in the management (14).

Antibiotic therapy is another important aspect that should be considered in DFU treatment (15). This is aimed at treating infection and not at healing the wound. It is crucial to investigate whether an infection is present according to the International Working Group on the Diabetic foot (IWGDF) guidelines. Empirical antibiotic treatment is often performed and should be based on a clinical suspicion of the causative bacteria, clinical severity, presence of previous microbiological culture results, presence of comorbidities such as chronic kidney failure, and antibiotic allergy history (16). If possible, a deep-wound surgical specimen should be obtained (7). Antibiotic treatment must be provided, with a narrow spectrum tailored to the microbiological results and the duration discussed by infectious disease specialists.

## 2 Diabetic complications

Diabetes mellitus is associated with various vascular complications that have traditionally been divided into two categories: macrovascular and microvascular pathologies. Macrovascular conditions include coronary heart disease, peripheral arterial disease (PAD), and stroke, while microvascular diseases include retinopathy, diabetic kidney disease (DKD), and peripheral neuropathy. These complications are very common, as approximately half of the individuals with diabetes have microvascular complications and more than a quarter have macrovascular complications (17). In diabetes, increased all-cause mortality rate is associated with cardiovascular, cerebrovascular, and chronic kidney diseases. According to the International Diabetes Federation, 6.7 million deaths can be attributed to diabetes in 2021 (18).

Other medical conditions commonly associated with diabetes include dementia, cancer, nonalcoholic fatty liver disease, and obstructive sleep apnea (19). These conditions now play a major role in the diabetes-related morbidity and mortality. For example, cancer is now considered the primary cause of death among individuals with diabetes in some countries, and the number of deaths attributed to dementia has significantly increased over the past several decades (20, 21). Owing to the many complications associated with diabetes, significant morbidity has been observed, resulting in an estimated 68 million disability-adjusted life years (22).

## 3 Diabetic foot disorders and amputations

**Figure 1:**
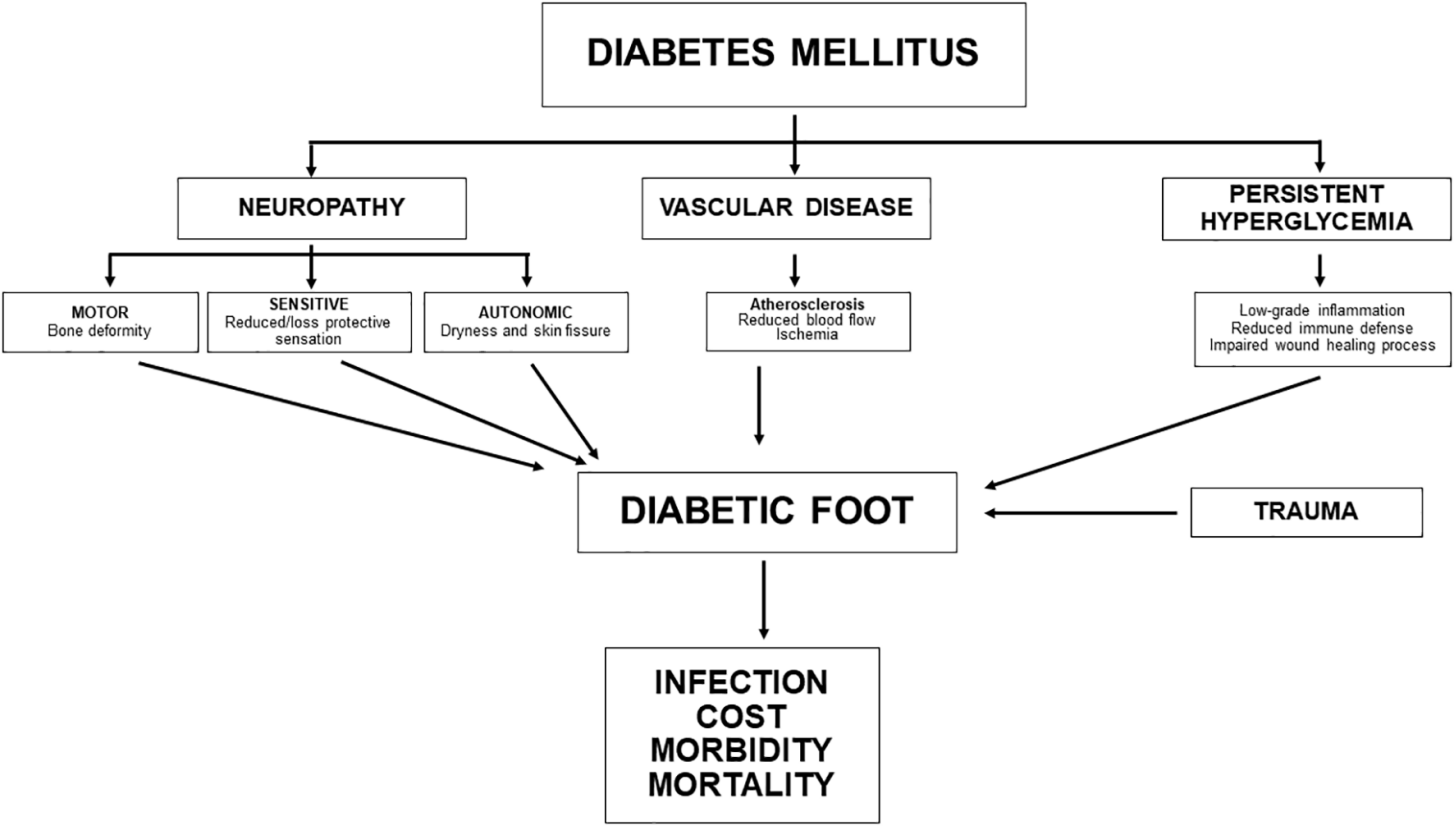
Pathophysiology of diabetic foot disorderss.

Diabetic foot is a major complication of diabetes and includes a spectrum of injuries such as ulceration, infection, and destruction of tissue or bone. It is practically always the result of microvascular damage with neuropathy and/or macrovascular damage in the form of PAD (**Figure 1**). Diabetes is a serious and potentially devastating complication. The annual incidence of DFU is estimated to be less than 2.2%, and the risk of developing foot ulcers over the course of the life of a person with diabetes is greater than 30%. The rate of recurrence after wound healing reaches approximately 40% in the year after the episode (23). A meta-analysis of 67 studies mainly from Europe and Asia, including 801,985 individuals, showed a variation in its prevalence between 3% in Oceania and 13% in North America, with a global average of 6.3% (24). Owing to the increasing prevalence of diabetes and prolonged life expectancy, the incidence of DFU is expected to increase in parallel.

For many vascular diseases, the most established risk factors for DFU are male sex, diabetes duration, HbA1c level, active smoking, high body mass index, PAD, and chronic kidney disease (24). Proinflammatory cytokine levels were also significantly higher in patients with diabetes with DFU than in those without DFU (25).

In the context of diabetic foot, adiponectin appears to promote wound healing as well as to protect against the development of atherosclerotic plaques and, therefore, cardiovascular (CV) diseases (26, 27). Its potential therapeutic role in diabetes, more specifically in diabetic foot, remains to be determined. Altogether, these findings highlight that diabetic foot is characterized by an exacerbated inflammatory state that promotes atherosclerosis; therefore, CV events contribute to the associated mortality in DFU. The increased CV risk in people with diabetes and DFU can be attributed, at least partially, to biochemical alterations, such as serum LDL > 130 mg/dL, hypertriglyceridemia, microalbuminuria, and proteinuria (28).

Peripheral arterial disease is an atherosclerotic narrowing of the peripheral arteries of the lower extremities and another contributor to DFU. The main risk factors of PAD are diabetes, hypertension, dyslipidemia, smoking, and age. Patients with diabetes with PAD are generally unaware of their condition, and signs and symptoms often only appear when the disease has already advanced, indicating that PAD remains largely underdiagnosed. When present, the signs and symptoms of PAD include pain in the lower limbs on exertion or at rest, non-healing wounds, ulcers, or gangrenes (29). The prevalence of PAD in patients with DFU is > 40% (30). The cornerstone of PAD management is revascularization of narrowed or occluded lower limb arteries to restore blood flow and induce wound healing. In PAD, the presence of diabetes is associated with more severe and distal arterial lesions, as well as a higher rate of amputation and mortality, compared with the absence of diabetes (31). In a series of 583 patients who underwent minor amputations due to diabetic foot osteomyelitis, 84% of those who required transtibial amputation over the follow-up period had concomitant PAD (32).

Diabetic foot ulceration generally develops after repetitive or minor (unrecognized) trauma to a part of the foot, with impaired wound healing due to PAD and/or peripheral neuropathy. Diabetic peripheral neuropathy (DPN) is the main cause of DFU. DPN can be sensitive, motor, or autonomic in nature. Sensitive neuropathy reduces the protective sensation of the feet, thereby reducing the detection of minor trauma, thermal injury, or overpressure in certain areas of the foot. Diabetic peripheral sensitive neuropathy is the primary component in more than half of all diabetic foot disorders (33). Motor neuropathy affects the biochemical aspects of foot ulceration, with progressive foot structural alteration leading to joint mobility impairment and anatomical deformation, causing detrimental and inappropriate pressure loads on the foot (34). Consequently, calluses appear on an area under high pressure, which promotes skin cracking, with perforation of the subcutaneous tissue contributing to DFU formation. Diabetic autonomic neuropathy elicits dryness, alterations in skin texture, edema, venous prominence, and nail loss, thereby leading to the development of DFU (35). Approximately half of all diabetic foot disorders are complicated by diabetic foot infection (DFI), and approximately 20% of moderate or severe infection episodes eventually require lower-extremity amputations at various levels (23). One in five diabetic food disorders is associated with the presence of (chronic) osteomyelitis (36).

Diabetic foot infection is a leading cause of hospitalization among people with diabetes, representing 20% of hospital admissions in the United States of America (USA). DFI occurs mostly due to terminal ischemia that is not amenable to revascularization and slightly due to infection. However, infection can precipitate the need for the amputation of a chronically ischemic foot. Additionally, DFI is associated with a readmission rate of approximately 40% (37, 38). Diabetes-related lower extremity amputation is preceded in most cases by DFU, which can be associated with ischemia or infection and is the number one cause of non-traumatic lower limb amputations worldwide.

Amputation is often considered the last option for the management of non-salvageable limbs. The indications for this procedure are the presence of extensive necrotic tissues with possible rapid extension and several conditions for which the patient and clinician are of the opinion that amputation will yield better results in terms of the overall improvement in locomotor function and quality of life (39). The selection of the amputation procedure type depends on the extent of bone infection, degree of lower limb arterial insufficiency, severity of soft tissue injury, and patient’s overall clinical state and functionality (40). The pursuit of antibiotics after amputation for an episode of DFI may not be needed (41). Globally, every 30 s, diabetes-related lower limb amputation is performed (42). It is estimated that approximately 90% and 67% of yearly amputations in the United Kingdom and USA, respectively, are related to diabetes (43). An episode of lower limb amputation should be considered a major risk factor for subsequent amputations as illustrated in a series of 102 individuals with transtibial amputation. In this cohort, at 2 years of follow-up after amputation, one-third of patients developed a diabetic foot disorder in the contralateral limb, and 10% underwent contralateral transtibial amputation (44). A series of 583 cases of diabetic foot osteomyelitis identified hind foot localization as an independent risk factor for limb loss (odds ratio, 5.4) (32).

## 4 Morbidity related to diabetic foot disorders

Diabetic foot disorders are associated with substantial morbidity and significantly reduced health-related quality of life (HRQoL) (42). They represent a considerable and increasingly prominent component of the global disability burden, with more than 2% of the global years lived with disability and almost two-thirds of all diabetes-related years lived with disability (45). A meta-analysis of 12 studies showed that people with DFU have significantly reduced HRQoL, especially in terms of their physical capacity and perception of general health (46).

Reduced HRQoL has been reported not only in patients with active ulcers and previous episodes of amputation but also in people whose ulcers have healed. A study of more than 300 participants with previous foot ulceration reported reduced HRQoL in most indicators, with the physical domain showing the greatest reduction in these patients compared with the control population (47).

In a study by the Eurodiale group, healing of DFU was reported to be associated with improvement in HRQoL. Another study that prospectively assessed the impact of hyperbaric oxygen therapy revealed a significantly higher mental quality of life, better social function, and reduced physical limitations in individuals who were healed compared with those who were not healed (48).

Functional recovery at the locomotor level in the postoperative period is crucial for a patient’s quality of life and overall health. Indeed, after transtibial amputation, patients can be ambulated based on a perception of superior well-being as well as the fact that being ambulatory is associated with improvement in cardiovascular health, which can reduce morbidity and mortality (49). One disadvantage of the multidisciplinary management of DFU is that patients are required to attend multiple appointments with various healthcare workers, which may be considered burdensome. Altogether, diabetic foot disease is associated with low HRQoL, particularly with regard to the physical quality of life. However, currently, no gold standard tool exists to assess patient-reported outcomes in diabetic foot disorders, and healing of diabetic foot ulcers is ultimately associated with improvement in quality of life (50).

## 5 Mortality related to diabetic foot disorders

The 5-year overall mortality in individuals with DFU or diabetic Charcot arthropathy is approximately 30% and increases to more than 50% after a major amputation. Therefore, the survival rate after a major amputation is lower than the 5-year survival rate of patients with most local cancers (51). The risk factors associated with mortality in DFU are age, male sex, chronic kidney disease, and presence of PAD. Compared with that of patients of the same age and disease, the life expectancy of those with DFU is lower by five years (52, 53).

In addition, people with DFU or diabetic Charcot neuroarthropathy have an estimated reduction in life expectancy of 14 years (54). The severity of the level of amputation is correlated with the survival rate as shown in a study reporting a two-fold higher 2-year survival rate in patients with minor amputation (below the ankle) than in those with transtibial amputation (54).

The increased mortality risk in patients with DFU is primarily due to cardiovascular events. Patients with DFU have more CV risk factors, CV pathologies, and subclinical markers of CV diseases, compared with patients with diabetes without DFU (55). Patients with diabetic neuropathy display increased levels of inflammatory cytokines such as TNF-a, IL-1, and IL-6 (56).

Therefore, diabetic foot disease is associated with a high rate of mortality, although for patients, the fear of a major amputation remains higher than the risk of death (57). This highlights the fact that a significant proportion of mortality in diabetic foot complications is related to associated comorbidities, such as cardiovascular disease and DKD. However, DFU itself has been shown to be an independent risk factor for premature mortality and lower limb amputation (58).

These observations have led to the suggestion of terminology modification in DFU by preferentially using the term “in remission” instead of “healed” to better illustrate the significant risk of recurrence for the patients and physicians and, therefore, the importance of regular and life-time follow-up after any episode of DFU (51).

## 6 General costs related to diabetes and diabetic foot disorders

Diabetes mellitus has incurred at least USD 966 billion in health expenditures worldwide, with a 316% increase over the past 15 year (18). Diabetic foot complications have major economic impacts on patients, families, and society. Information regarding expenditures related to diabetic foot is of particular importance, especially for public health policymakers, to encourage and develop prevention and therapeutic programs dedicated to this disorder. To properly assess the economic burden of diabetic foot diseases, it is crucial to consider direct and indirect costs such as primary care, podiatrist care, nursing care, special footwear, hospitalization, rehabilitation medicine, consequences of lower limb amputations, including loss of productivity, home care, family status and costs, and reduced quality of life (**Table 1**). Therefore, comparisons of the costs associated with diabetic foot between different areas of the world or countries remain complex, as the health economic machinery, including the reimbursement system, is not uniform. Furthermore, treatment approaches, reimbursement policies, and study designs remain heterogeneous among the published and available data worldwide. The severity of diabetic foot disorders is correlated with the associated costs. Indeed, expenditure related to DFU is higher if the severity of the ulcer is higher, and healthcare expenditures are five times higher in people with diabetes with DFU than in those without ulcer (59). Therefore, expenditures related to diabetic foot disease may vary greatly depending on the interventions used and the overall management approach. In Europe, the annual direct and indirect costs related to DFU are estimated at approximately USD 13 500 per person affected. In the UK, approximately 0.6% of the National Health Service budget is allocated to cover the management of DFU (60).

**Table 1:** Direct and indirect cost associated with diabetic foot disorders.

Direct cost	
Primary care
Global diabetes management including glucose control
Medical and nursing ambulatory care
Podiatrist associated care
Wound dressing
Debridement
Antibiotics
Microbiological analysis
Offloading devices
orthopedic diabetic shoes
Radiological imaging
Revascularization procedure
Orthopedic surgery
Amputation
Hospitalization
Physiotherapy
Orthopedic prostheses

Indirect cost	
Loss of productivity
Under employment/unemployment
Absenteeism
Reduce family asset
Early retirement
Transportation
Psychotherapy
Home care

There are several studies that have evaluated the costs with the management of DFU. These costs vary greatly depending on the regions of the world and the periods during which the studies were carried out (**Table 2**).

**Table 2:** Studies reporting the cost of diabetic foot.

Authors, year	Country	Number of individuals	Type of care	Cost
Alshammary, 2020 (61)	Saudi Arabia	99	Episode of DFU	1783 USD
Apelqivst, 1994 (62)	Sweden	197	All ulcer types; total direct costs	8659 USD
Ashry, 1998 (63)	USA	5062	Lower-extremity amputations, hospital charges only	27930 USD
Danmusa UM, 2016 (64)	Nigeria	1573	Episode of DFU	1104 USD
Girod, 2003 (65)	France	239	Monthly healthcare expenditure for DFU	1265 USD
Greenidge, 2021 (66)	Barbados	50	Episode of DFU	7272 USD
Jodheea-Jutton, 2022 (67)	India	7487	Episode of DFU	1960 USD
Kerr, 2014 (60)	The UK	72 459	Per episode of DFU	7539 USD
Lo, 2021 (68)	Singapore	1729	Mean cost per patient-year	3368 USD
Lu, 2020 (69)	China	3654	Episode of DFU	¥21827
Oksuz, 2016 (70)	Turkey	5000	Out-patient and in-patients costs of DFU care	14 288 USD
Prompers, 2007 (59)	Nine European countries	821	DFUs, total annual total direct and indirect cost	10,091 Euros
Toscano, 2018 (71)	Brazil	12994	Inpatient cost of DFU care	306 USD
Van Acker, 2000 (72)	Belgium	167	Direct and indirect expenditure per episode of DFU	10 572 USD

USD, United States dollar; ¥, Yen.

In the United States of America, the direct expenditure attributed to diabetes was estimated at USD 237 billion in 2017, representing an increase of more than 25% from 2012, including 30% related to diabetic foot management. This cost is in the same range as that of cancer in 2015 (USD 80 billion) (51). In developing countries, the available evidence of expenditures related to diabetic foot remains limited, even though its burden is most likely to be higher. In Brazil, one study estimated the direct medical costs of inpatient and outpatient care for diabetic foot disorders. The annual cost represented 0.3% of all public health expenditures and mainly comprised outpatient care rather than inpatient care (87% vs. 13%, respectively) (71). It remains very difficult to correctly estimate the unrelated costs of transport, which vary according to different factors such as area, medical density and the literature currently available is limited. Transport is a real barrier for patients, especially those living in rural areas but also in urban areas, Due in particular to traffic jams, parking problems, travel fatigue and access limitations when using wheelchairs (73). Telemedicine could represent a step forward in this aspect and make it possible to reduce transport-related contracts. A recent randomized study showed that in patients with DFU, the addition of monitoring by telemedicine with a specialized nurse makes it possible to reduce costs and hospital stays compared to conventional monitoring and also probably indirectly costs and transport constraints (74). DFU is associated with a high emotional load, favoring anxiety and depression (75). The presence of depression will not only necessitate costly psychological support, but will also have an impact on quality of life by reducing it. The psychological cost associated with DFU could be considered an indirect cost of care, but is not yet clearly evaluated and estimated in the literature, and would therefore require future analysis. Reduced mobility and performance in the workplace will lead to frustration and emotional distress, making it even more difficult to provide comprehensive care (76). The loss of productivity and the risk of unemployment and/or early retirement may have also significant impact on the family asset, but remain to be properly quantified (76).

Therefore, preventive approaches to reduce DFU and lower limb amputation are considered a fundamental way to decrease the high costs associated. Available and regular diabetic foot care is a major way to decrease amputation rates in individuals with diabetes. A multi-disciplinary team approach remains crucial, as it has been shown to decrease amputation rates by approximately 85% and reduce the risks associated with DFU, leading to a higher quality of life (77, 78). The costs related to DFU are expected to increase alongside the prevalence of diabetes.

Future detection and implementation of cost-saving and cost-effective measures for the management of DFU are required to decrease its healthcare burden. Therefore, future research with economic comparisons of several strategies is required in the field of DFU.

## 7 Conclusions

In conclusion, diabetic foot disorders are associated with high rates of morbidity and mortality and have a major impact on health-related expenditures. As the prevalence of diabetes continues to increase, concerted efforts are needed in terms of prevention and treatment to reduce the burden associated with its complications, such as diabetic foot disorders. There is still ample scope for improving the current organization of available preventive and therapeutic care for diabetic foot disorders in various areas of the world. Therefore, new global strategies are urgently needed to counteract the deleterious effect of diabetes.

## Author contributions

FW: Writing – review & editing. IU: Writing – review & editing. LS-B: Writing – review & editing. CS: Writing – review & editing. KG: Conceptualization, Writing – original draft, Writing – review & editing.

## References

[1] Sun H Saeedi P Karuranga S Pinkepank M Ogurtsova K Duncan BB. IDF Diabetes Atlas: Global, regional and country-level diabetes prevalence estimates for 2021 and projections for 2045. Diabetes Res Clin Pract (2022) 183:109119. doi: 10.1016/j.diabres.2021.109119 34879977 PMC11057359

[2] Yang JJ Yu D Wen W Saito E Rahman S Shu XO. Association of diabetes with all-cause and cause-specific mortality in asia: A pooled analysis of more than 1 million participants. JAMA Netw Open (2019) 2(4):e192696. doi: 10.1001/jamanetworkopen.2019.2696 31002328 PMC6481439

[3] Salehidoost R Mansouri A Amini M Aminorroaya Yamini S Aminorroaya A. Diabetes and all-cause mortality, a 18-year follow-up study. Sci Rep (2020) 10(1):3183. doi: 10.1038/s41598-020-60142-y 32081921 PMC7035261

[4] Bonnet JB Sultan A. Social deprivation, healthcare access and diabetic foot ulcer: A narrative review. J Clin Med (2022) 11(18):1–10. doi: 10.3390/jcm11185431 PMC9501414 36143078

[5] Crocker RM Palmer KNB Marrero DG Tan TW. Patient perspectives on the physical, psycho-social, and financial impacts of diabetic foot ulceration and amputation. J Diabetes Complications. (2021) 35(8):107960. doi: 10.1016/j.jdiacomp.2021.107960 34059410 PMC8316286

[6] Uzzaman MM Jukaku S Kambal A Hussain ST. Assessing the long-term outcomes of minor lower limb amputations: a 5-year study. Angiology (2011) 62(5):365–71. doi: 10.1177/0003319710395558 21421619

[7] Lipsky BA Senneville E Abbas ZG Aragon-Sanchez J Diggle M Embil JM. Guidelines on the diagnosis and treatment of foot infection in persons with diabetes (IWGDF 2019 update). Diabetes Metab Res Rev (2020) 36 Suppl 1:e3280. doi: 10.1002/dmrr.3280 32176444

[8] Hap K Biernat K Konieczny G. Patients with diabetes complicated by peripheral artery disease: the current state of knowledge on physiotherapy interventions. J Diabetes Res (2021) 2021:5122494. doi: 10.1155/2021/5122494 34056006 PMC8131145

[9] Meloni M Andreadi A Bellizzi E Giurato L Ruotolo V Romano M. A multidisciplinary team reduces in-hospital clinical complications and mortality in patients with diabetic foot ulcers. Diabetes Metab Res Rev (2023) 39(7):e3690. doi: 10.1002/dmrr.3690 37422897

[10] Musuuza J Sutherland BL Kurter S Balasubramanian P Bartels CM Brennan MB. A systematic review of multidisciplinary teams to reduce major amputations for patients with diabetic foot ulcers. J Vasc Surg (2020) 71(4):1433–46 e3. doi: 10.1016/j.jvs.2019.08.244 31676181 PMC7096268

[11] Bus SA Armstrong DG Crews RT Gooday C Jarl G Kirketerp-Moller K. Guidelines on offloading foot ulcers in persons with diabetes (IWGDF 2023 update). Diabetes Metab Res Rev (2023):e3647. doi: 10.1002/dmrr.3647 37226568

[12] Marston WA Dermagraft Diabetic Foot Ulcer Study G. Risk factors associated with healing chronic diabetic foot ulcers: the importance of hyperglycemia. Ostomy Wound Manage (2006) 52(3):26–8. 16567857

[13] Davies MJ Aroda VR Collins BS Gabbay RA Green J Maruthur NM. Management of hyperglycemia in type 2 diabetes, 2022. A consensus report by the american diabetes association (ADA) and the european association for the study of diabetes (EASD). Diabetes Care (2022) 45(11):2753–86. doi: 10.2337/dci22-0034 PMC10008140 36148880

[14] Alvaro-Afonso FJ Lazaro-Martinez JL Papanas N. To smoke or not to smoke: cigarettes have a negative effect on wound healing of diabetic foot ulcers. Int J Low Extrem Wounds. (2018) 17(4):258–60. doi: 10.1177/1534734618808168 30760072

[15] Uckay I Gariani K Dubois-Ferriere V Suva D Lipsky BA. Diabetic foot infections: recent literature and cornerstones of management. Curr Opin Infect Dis (2016) 29(2):145–52. doi: 10.1097/QCO.0000000000000243 26779774

[16] Gariani K Lebowitz D Kressmann B von Dach E Sendi P Waibel F. Oral amoxicillin-clavulanate for treating diabetic foot infections. Diabetes Obes Metab (2019) 21(6):1483–6. doi: 10.1111/dom.13651 30719838

[17] Litwak L Goh SY Hussein Z Malek R Prusty V Khamseh ME. Prevalence of diabetes complications in people with type 2 diabetes mellitus and its association with baseline characteristics in the multinational A1chieve study. Diabetol Metab Syndr (2013) 5(1):57. doi: 10.1186/1758-5996-5-57 24228724 PMC3854020

[18] Miller EM. Using continuous glucose monitoring in clinical practice. Clin Diabetes. (2020) 38(5):429–38. doi: 10.2337/cd20-0043 PMC7755046 33384468

[19] Tomic D Shaw JE Magliano DJ. The burden and risks of emerging complications of diabetes mellitus. Nat Rev Endocrinol (2022) 18(9):525–39. doi: 10.1038/s41574-022-00690-7 PMC9169030 35668219

[20] Pearson-Stuttard J Bennett J Cheng YJ Vamos EP Cross AJ Ezzati M. Trends in predominant causes of death in individuals with and without diabetes in England from 2001 to 2018: an epidemiological analysis of linked primary care records. Lancet Diabetes Endocrinol (2021) 9(3):165–73. doi: 10.1016/S2213-8587(20)30431-9 PMC7886654 33549162

[21] Pearson-Stuttard J Buckley J Cicek M Gregg EW. The changing nature of mortality and morbidity in patients with diabetes. Endocrinol Metab Clin North Am (2021) 50(3):357–68. doi: 10.1016/j.ecl.2021.05.001 34399950

[22] Lin X Xu Y Pan X Xu J Ding Y Sun X. Global, regional, and national burden and trend of diabetes in 195 countries and territories: an analysis from 1990 to 2025. Sci Rep (2020) 10(1):14790. doi: 10.1038/s41598-020-71908-9 32901098 PMC7478957

[23] Armstrong DG Boulton AJM Bus SA. Diabetic foot ulcers and their recurrence. N Engl J Med (2017) 376(24):2367–75. doi: 10.1056/NEJMra1615439 28614678

[24] Zhang P Lu J Jing Y Tang S Zhu D Bi Y. Global epidemiology of diabetic foot ulceration: a systematic review and meta-analysis (dagger). Ann Med (2017) 49(2):106–16. doi: 10.1080/07853890.2016.1231932 27585063

[25] Weigelt C Rose B Poschen U Ziegler D Friese G Kempf K. Immune mediators in patients with acute diabetic foot syndrome. Diabetes Care (2009) 32(8):1491–6. doi: 10.2337/dc08-2318 PMC2713614 19509015

[26] Shibata S Tada Y Asano Y Hau CS Kato T Saeki H. Adiponectin regulates cutaneous wound healing by promoting keratinocyte proliferation and migration via the ERK signaling pathway. J Immunol (2012) 189(6):3231–41. doi: 10.4049/jimmunol.1101739 22904306

[27] Freitas Lima LC Braga VA do Socorro de Franca Silva M Cruz JC Sousa Santos SH de Oliveira Monteiro MM. Adipokines, diabetes and atherosclerosis: an inflammatory association. Front Physiol (2015) 6:304. doi: 10.3389/fphys.2015.00304 26578976 PMC4630286

[28] Tuttolomondo A Maida C Pinto A. Diabetic foot syndrome as a possible cardiovascular marker in diabetic patients. J Diabetes Res (2015) 2015:268390. doi: 10.1155/2015/268390 25883983 PMC4391526

[29] American Diabetes A. Peripheral arterial disease in people with diabetes. Diabetes Care (2003) 26(12):3333–41. doi: 10.2337/diacare.26.12.3333 14633825

[30] Azhar A Basheer M Abdelgawad MS Roshdi H Kamel MF. Prevalence of peripheral arterial disease in diabetic foot ulcer patients and its impact in limb salvage. Int J Low Extrem Wounds. (2021) 22:518–23. doi: 10.1177/15347346211027063 34142882

[31] Abbott RD Brand FN Kannel WB. Epidemiology of some peripheral arterial findings in diabetic men and women: experiences from the Framingham Study. Am J Med (1990) 88(4):376–81. doi: 10.1016/0002-9343(90)90492-V 2327425

[32] Winkler E Schöni M Krähenbühl N Uçkay I Waibel FWA. Foot osteomyelitis location and rates of primary or secondary major amputations in patients with diabetes. Foot Ankle Int (2022) 43(7):957–67. doi: 10.1177/10711007221088552 PMC9260474 35582923

[33] Walters DP Gatling W Mullee MA Hill RD. The distribution and severity of diabetic foot disease: a community study with comparison to a non-diabetic group. Diabetes Med (1992) 9(4):354–8. doi: 10.1111/j.1464-5491.1992.tb01796.x 1600707

[34] Sutkowska E Sutkowski K Sokolowski M Franek E Dragan S. Distribution of the highest plantar pressure regions in patients with diabetes and its association with peripheral neuropathy, gender, age, and BMI: one centre study. J Diabetes Res (2019) 2019:7395769. doi: 10.1155/2019/7395769 31380446 PMC6652074

[35] Feldman EL Callaghan BC Pop-Busui R Zochodne DW Wright DE Bennett DL. Diabetic neuropathy. Nat Rev Dis Primers. (2019) 5(1):41. doi: 10.1038/s41572-019-0092-1 31197153

[36] Senneville E Lipsky BA Abbas ZG Aragon-Sanchez J Diggle M Embil JM. Diagnosis of infection in the foot in diabetes: a systematic review. Diabetes Metab Res Rev (2020) 36 Suppl 1:e3281. doi: 10.1002/dmrr.3281 32176440

[37] Fincke BG Miller DR Turpin R. A classification of diabetic foot infections using ICD-9-CM codes: application to a large computerized medical database. BMC Health Serv Res (2010) 10:192. doi: 10.1186/1472-6963-10-192 20604921 PMC2914721

[38] Frykberg RG Wittmayer B Zgonis T. Surgical management of diabetic foot infections and osteomyelitis. Clin Podiatr Med Surg (2007) 24(3):469–82. doi: 10.1016/j.cpm.2007.04.001 17613386

[39] Rathnayake A Saboo A Malabu UH Falhammar H. Lower extremity amputations and long-term outcomes in diabetic foot ulcers: A systematic review. World J Diabetes. (2020) 11(9):391–9. doi: 10.4239/wjd.v11.i9.391 PMC7503503 32994867

[40] Primadhi RA Septrina R Hapsari P Kusumawati M. Amputation in diabetic foot ulcer: A treatment dilemma. World J Orthop (2023) 14(5):312–8. doi: 10.5312/wjo.v14.i5.312 PMC10251268 37304194

[41] Rossel A Lebowitz D Gariani K Abbas M Kressmann B Assal M. Stopping antibiotics after surgical amputation in diabetic foot and ankle infections-A daily practice cohort. Endocrinol Diabetes Metab (2019) 2(2):e00059. doi: 10.1002/edm2.59 31008367 PMC6458464

[42] Boulton AJ Vileikyte L Ragnarson-Tennvall G Apelqvist J. The global burden of diabetic foot disease. Lancet (2005) 366(9498):1719–24. doi: 10.1016/S0140-6736(05)67698-2 16291066

[43] Ahmad N Thomas GN Gill P Torella F. The prevalence of major lower limb amputation in the diabetic and non-diabetic population of England 2003-2013. Diabetes Vasc Dis Res (2016) 13(5):348–53. doi: 10.1177/1479164116651390 27334482

[44] Wukich DK Ahn J Raspovic KM Gottschalk FA La Fontaine J Lavery LA. Comparison of transtibial amputations in diabetic patients with and without end-stage renal disease. Foot Ankle Int (2017) 38(4):388–96. doi: 10.1177/1071100716688073 28103735

[45] Zhang Y Lazzarini PA McPhail SM van Netten JJ Armstrong DG Pacella RE. Global disability burdens of diabetes-related lower-extremity complications in 1990 and 2016. Diabetes Care (2020) 43(5):964–74. doi: 10.2337/dc19-1614 32139380

[46] Khunkaew S Fernandez R Sim J. Health-related quality of life among adults living with diabetic foot ulcers: a meta-analysis. Qual Life Res (2019) 28(6):1413–27. doi: 10.1007/s11136-018-2082-2 30565072

[47] Perrin BM van Netten JJ Aan de Stegge WB Busch-Westbroek TE Bus SA. Health-related quality of life and associated factors in people with diabetes at high risk of foot ulceration. J Foot Ankle Res (2022) 15(1):83. doi: 10.1186/s13047-022-00586-9 36401293 PMC9675249

[48] Siersma V Thorsen H Holstein PE Kars M Apelqvist J Jude EB. Diabetic complications do not hamper improvement of health-related quality of life over the course of treatment of diabetic foot ulcers - the Eurodiale study. J Diabetes Complications. (2017) 31(7):1145–51. doi: 10.1016/j.jdiacomp.2017.04.008 28457703

[49] Wukich DK Ahn J Raspovic KM La Fontaine J Lavery LA. Improved quality of life after transtibial amputation in patients with diabetes-related foot complications. Int J Low Extrem Wounds. (2017) 16(2):114–21. doi: 10.1177/1534734617704083 28682728

[50] Wukich DK Raspovic KM. Assessing health-related quality of life in patients with diabetic foot disease: why is it important and how can we improve? The 2017 roger E. Pecoraro award lecture. Diabetes Care (2018) 41(3):391–7. doi: 10.2337/dci17-0029 29463665

[51] Armstrong DG Swerdlow MA Armstrong AA Conte MS Padula WV Bus SA. Five year mortality and direct costs of care for people with diabetic foot complications are comparable to cancer. J Foot Ankle Res (2020) 13(1):16. doi: 10.1186/s13047-020-00383-2 32209136 PMC7092527

[52] Ghanassia E Villon L Thuan Dit Dieudonne JF Boegner C Avignon A Sultan A. Long-term outcome and disability of diabetic patients hospitalized for diabetic foot ulcers: a 6.5-year follow-up study. Diabetes Care (2008) 31(7):1288–92. doi: 10.2337/dc07-2145 PMC2453665 18390801

[53] Morbach S Furchert H Groblinghoff U Hoffmeier H Kersten K Klauke GT. Long-term prognosis of diabetic foot patients and their limbs: amputation and death over the course of a decade. Diabetes Care (2012) 35(10):2021–7. doi: 10.2337/dc12-0200 PMC3447849 22815299

[54] van Baal J Hubbard R Game F Jeffcoate W. Mortality associated with acute Charcot foot and neuropathic foot ulceration. Diabetes Care (2010) 33(5):1086–9. doi: 10.2337/dc09-1428 PMC2858181 20185744

[55] Pinto A Tuttolomondo A Di Raimondo D Fernandez P La Placa S Di Gati M. Cardiovascular risk profile and morbidity in subjects affected by type 2 diabetes mellitus with and without diabetic foot. Metabolism (2008) 57(5):676–82. doi: 10.1016/j.metabol.2008.01.004 18442633

[56] Herder C Lankisch M Ziegler D Rathmann W Koenig W Illig T. Subclinical inflammation and diabetic polyneuropathy: MONICA/KORA Survey F3 (Augsburg, Germany). Diabetes Care (2009) 32(4):680–2. doi: 10.2337/dc08-2011 PMC2660451 19131463

[57] Wukich DK Raspovic KM Suder NC. Patients with diabetic foot disease fear major lower-extremity amputation more than death. Foot Ankle Spec. (2018) 11(1):17–21. doi: 10.1177/1938640017694722 28817962

[58] Martins-Mendes D Monteiro-Soares M Boyko EJ Ribeiro M Barata P Lima J. The independent contribution of diabetic foot ulcer on lower extremity amputation and mortality risk. J Diabetes Complications. (2014) 28(5):632–8. doi: 10.1016/j.jdiacomp.2014.04.011 PMC4240944 24877985

[59] Prompers L Huijberts M Schaper N Apelqvist J Bakker K Edmonds M. Resource utilisation and costs associated with the treatment of diabetic foot ulcers. Prospective data from the Eurodiale Study. Diabetologia (2008) 51(10):1826–34. doi: 10.1007/s00125-008-1089-6 18648766

[60] Kerr M Rayman G Jeffcoate WJ. Cost of diabetic foot disease to the National Health Service in England. Diabetes Med (2014) 31(12):1498–504. doi: 10.1111/dme.12545 24984759

[61] Alshammary. Ann Saudi Med (2020) 40(5):425–35. doi: 10.5144/0256-4947.2 PMC7532050 33007171

[62] Apelqvist J Ragnarson-Tennvall G Persson U Larsson J. Diabetic foot ulcers in a multidisciplinary setting. An economic analysis of primary healing and healing with amputation. J Intern Med (1994) 235(5):463–71. doi: 10.1111/j.1365-2796.1994.tb01104.x 8182403

[63] Ashry HR Lavery LA Armstrong DG Lavery DC van Houtum WH. Cost of diabetes-related amputations in minorities. J Foot Ankle Surg (1998) 37(3):186–90. doi: 10.1016/S1067-2516(98)80108-7 9638541

[64] Danmusa UM Terhile I Nasir IA Ahmad AA Muhammad HY. Prevalence and healthcare costs associated with the management of diabetic foot ulcer in patients attending Ahmadu Bello University Teaching Hospital, Nigeria. Int J Health Sci (Qassim). (2016) 10(2):219–28. doi: 10.12816/0048814 PMC4825895 27103904

[65] Girod I Valensi P Laforet C Moreau-Defarges T Guillon P Baron F. An economic evaluation of the cost of diabetic foot ulcers: results of a retrospective study on 239 patients. Diabetes Metab (2003) 29(3):269–77. doi: 10.1016/S1262-3636(07)70036-8 12909815

[66] Greenidge AR Quimby KR Rose AMC Speede A Hambleton IR Anderson SG. Direct healthcare services cost of non-healing diabetic foot wounds in an African origin population in Barbados. Diabetes Med (2022) 39(6):e14773. doi: 10.1111/dme.14773 34936707

[67] Jodheea-Jutton A Hindocha S Bhaw-Luximon A. Health economics of diabetic foot ulcer and recent trends to accelerate treatment. Foot (Edinb). (2022) 52:101909. doi: 10.1016/j.foot.2022.101909 36049265

[68] Lo ZJ Surendra NK Saxena A Car J. Clinical and economic burden of diabetic foot ulcers: A 5-year longitudinal multi-ethnic cohort study from the tropics. Int Wound J (2021) 18(3):375–86. doi: 10.1111/iwj.13540 PMC8244009 33497545

[69] Lu Q Wang J Wei X Wang G Xu Y Lu Z. Cost of diabetic foot ulcer management in China: A 7-year single-center retrospective review. Diabetes Metab Syndr Obes (2020) 13:4249–60. doi: 10.2147/DMSO.S275814 PMC7667006 33204131

[70] Oksuz E Malhan S Sonmez B Numanoglu Tekin R. Cost of illness among patients with diabetic foot ulcer in Turkey. World J Diabetes. (2016) 7(18):462–9. doi: 10.4239/wjd.v7.i18.462 PMC5065666 27795820

[71] Toscano CM Sugita TH Rosa MQM Pedrosa HC Rosa RDS Bahia LR. Annual direct medical costs of diabetic foot disease in Brazil: A cost of illness study. Int J Environ Res Public Health (2018) 15(1):1–13. doi: 10.3390/ijerph15010089 PMC5800188 29316689

[72] Van Acker K Oleen-Burkey M De Decker L Vanmaele R Van Schil P Matricali G. Cost and resource utilization for prevention and treatment of foot lesions in a diabetic foot clinic in Belgium. Diabetes Res Clin Pract (2000) 50(2):87–95. doi: 10.1016/S0168-8227(00)00157-1 10960718

[73] McPherson M Carroll M Stewart S. Patient-perceived and practitioner-perceived barriers to accessing foot care services for people with diabetes mellitus: a systematic literature review. J Foot Ankle Res (2022) 15(1):92. doi: 10.1186/s13047-022-00597-6 36527060 PMC9755774

[74] Dardari D Franc S Charpentier G Orlando L Bobony E Bouly M. Hospital stays and costs of telemedical monitoring versus standard follow-up for diabetic foot ulcer: an open-label randomised controlled study. Lancet Reg Health Eur (2023) 32:100686. doi: 10.1016/j.lanepe.2023.100686 37520145 PMC10384180

[75] Polikandrioti M Vasilopoulos G Koutelekos I Panoutsopoulos G Gerogianni G Alikari V. Depression in diabetic foot ulcer: Associated factors and the impact of perceived social support and anxiety on depression. Int Wound J (2020) 17(4):900–9. doi: 10.1111/iwj.13348 PMC7948719 32219987

[76] Palmer KNB Crocker RM Marrero DG Tan TW. A vicious cycle: employment challenges associated with diabetes foot ulcers in an economically marginalized Southwest US sample. Front Clin Diabetes Healthc. (2023) 4:1027578. doi: 10.3389/fcdhc.2023.1027578 37124466 PMC10140327

[77] Krishnan S Nash F Baker N Fowler D Rayman G. Reduction in diabetic amputations over 11 years in a defined U.K. population: benefits of multidisciplinary team work and continuous prospective audit. Diabetes Care (2008) 31(1):99–101. doi: 10.2337/dc07-1178 17934144

[78] Rubio JA Aragon-Sanchez J Jimenez S Guadalix G Albarracin A Salido C. Reducing major lower extremity amputations after the introduction of a multidisciplinary team for the diabetic foot. Int J Low Extrem Wounds. (2014) 13(1):22–6. doi: 10.1177/1534734614521234 24659624

